# Analysis of heavy metal contamination in topsoils across land use types within the Manghe River watershed in South Taihang and its source attribution

**DOI:** 10.1371/journal.pone.0335016

**Published:** 2025-10-17

**Authors:** Xiaoqiang Wan, Chengyu Wang, Quanlai Ma, Chongke Yang, Jizhou Zhang, Yingtao Shang

**Affiliations:** 1 The First Institute of Henan Provincial Resource Environment Survey, Zhengzhou, Henan Province, China; 2 School of Public Administration and Law, Northeast Agricultural University, Harbin, Heilongjiang Province, China; University of Technology Sydney, AUSTRALIA

## Abstract

To investigate the characteristics of soil heavy metal pollution in the Manghe River watershed, a typical industrial and mining complex area in the Yellow River Basin, concentrations of Hg, Cr, Cu, Ni, Pb, Zn, Cd, and pH were measured in 121 topsoil samples (0–20 cm) collected from the study area. Geostatistical methods were employed to analyze the spatial distribution patterns of heavy metals. The pollution status was assessed using the pollution load index (PLI), while correlation analysis, principal component analysis (PCA), and a positive matrix factorization (PMF) model were applied to identify the sources of heavy metals. The results indicated that: (1) The concentrations of Hg, As, Ni, Cu, Pb, Zn, and Cd exceeded their respective background values, with Hg, Pb and Cd reaching 3.52, 4.85, and 46.4 times of the background levels, respectively.(2) Different elements exhibited distinct spatial distribution and diffusion patterns, revealing their respective sources and influencing factors. (3) The overall PLI was 0.785, reflecting a mild pollution level across the region, while industrial and mining lands exhibited severe pollution (PLI = 4.3). The relative contribution of each heavy metal to the pollution load was ranked as follows: Cd (30.35)> Pb (4.76)> Hg (3.62)> Zn (2.18)> As (1.77)> Cu (1.53). (4) Principal component analysis categorized the sources of heavy metals into anthropogenic activities and natural origins. Further analysis using the PMF model delineated four specific sources: coal combustion (10.87%), natural and agricultural contributions (27.37%), transportation and agricultural actives (26.81%), and industrial emissions (34.95%). Finally, the study identified the following feasible strategies for controlling heavy metal pollution: blocking and remediating industrial pollution sources; treating agricultural non-point source pollution through biological methods; and substituting traditional transportation sources with new energy alternatives. This research could support decision-making processes related to the prevention and control of heavy metal pollution in the study area, as well as regional sustainable development.

## Introduction

Soil heavy metal pollution has emerged as one of the prominent global environmental pollution issues, with developing countries experiencing more severe impacts during rapid industrialization and urbanization [1,2]. As the largest developing country in the world, China also faces the pressing issue of soil heavy metal pollution. According to the National Soil Pollution Survey Bulletin jointly released by the Ministry of Ecology and Environment and the Ministry of Natural Resources in 2014, the exceedance rate of heavy metals in soil has reached 16.1%, and the situation is continuously worsening [3]. The 2022 China Ecological Environment Bulletin indicates that preventing and controlling soil heavy metal pollution remains a top priority. Heavy metal pollution is characterized by bioaccumulation, persistence, and concealment, making its remediation particularly challenging. Moreover, heavy metals can accumulate in soil, leading to ecological damage and entering the human body through food chain absorption or inhalation of dust particles, thereby posing a significant threat to human health [4,5]. Therefore, clarifying the distribution patterns and sources of heavy metal contamination in soil is of crucial importance for the prevention, control, and remediation of heavy metal pollution.

Regarding the spatial heterogeneity of soil heavy metal content across different land use types, the academic community has developed a multi-dimensional methodological framework. Traditional geostatistical approaches, such as Kriging interpolation and spatial autocorrelation analysis, are effective in revealing macro-level distribution patterns of heavy metals [6]; however, they fall short in explaining the underlying mechanisms of pollution formation, which are influenced by both anthropogenic activities and natural factors. Recently, GIS-based multi-model integration techniques have emerged to overcome these limitations. By combining spatial interpolation with pollution assessment, these techniques support an integrated analytical process-from spatial differentiation of pollution to land use response and finally to ecological risk classification [7,8]. In terms of pollution assessment, existing researches primarily employ five categories of indicator models: (1)Single-factor evaluation models, including the enrichment factor (EF) and the geo-accumulation index (Igeo), focus on threshold characteristics of individual elements [9,10]; (2) Composite index models, such as the Pollution Load Index (PLI) and Nemerow Index (NIPI), are suitable for assessing synergistic pollution involving multiple elements [11,12]; (3) Ecological risk indices, such as the Hakanson index, are used to quantify the biotoxic effects of heavy metals [13]; (4) Source-Sink relationship models, employing models like PMF and APCS-MLR, analyze pollutant migration pathways [14,15]; (5) Health risk assessment models establish Exposure Dose-Health Effect relationship.

For the scientific issue of pollution source analysis, research paradigms have evolved from the early qualitative identification of natural parent materials and human activities to more advanced quantitative attribution approaches [16]. The PMF model is particularly valuable for analyzing pollution sources in complex contaminated environments, such as industrial and agricultural areas, due to its its capabilities in quantifying uncertainty, enforcing non-negative constraints, and decomposing contributions from multiple sources [17]. Extensive researches show that the PMF model has exceptional capabilities when it comes to analyzing collinear pollution sources. These include industrial emissions (e.g., smelting dust and waste leaching [18]), agricultural non-point sources (e.g., heavy metal impurities in fertilizers and heavy metal accumulation from sewage irrigation [19]), and traffic-related sources (e.g., Zn from tyre wear and Cu from brake pads [20]). The deviation between the model’s calculated pollution source contribution rates and isotope tracing verification results can be controlled to within 15% [21].

This study examines the Manghe River Basin in Jiyuan City, a representative industrial and mining area within the Yellow River Basin. As a key industrial base under China’s “Central Region Rise” strategy, the region has established a comprehensive industrial system that includes steel smelting (accounting for 12% of the nation’s special steel production capacity), lead-zinc smelting (hosting Asia’s largest vertical-furnace zinc smelting base), and precious metal refining (producing 8% of the country’s silver output). Severe heavy metal pollution has resulted from the intertwined and overlapping processes of mining, mineral processing, smelting and manufacturing. The region urgently requires remediation of soil heavy metal pollution. In this study, we (1)combined statistics and geo-statistics methods to reveal the spatial heterogeneity of heavy metals, including Pb, Cr, Cd, Hg, As, Cu, Zn, and Ni; (2) used the Pollution Load Index (PLI) to quantify the contribution of pollution loads from different land use types; (3) applied a coupled PMF-PCA model to characterize source profiles of industrial, agricultural, and traffic emissions. Based on the results, a tiered control strategy is proposed, consisting of “interception and remediation of industrial sources, bio-interception of agricultural non-point sources, and new energy substitution for transportation sources.” This integrated framework of “spatial distribution analysis, pollution source identification, and remediation optimization” provides a systematic and practical approach for controlling soil heavy metal pollution in industrial and mining cities within the Yellow River Basin.

## Materials and methods

### Study area

**Fig 1:**
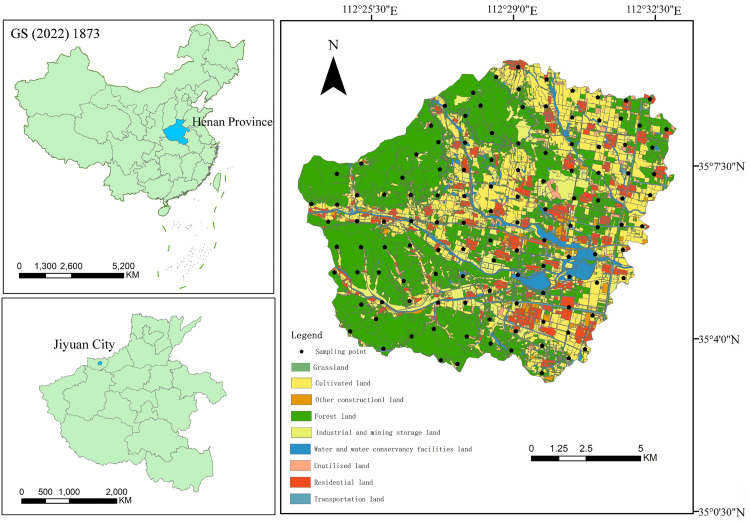
Land use type of sampling areas.

The Manghe River watershed is situated in a critical node of the ecological barrier of the Yellow River Basin. It spans from 112°23′37″ to 112°33′2″E and 35°3′4″ to 35°9′40″N (Fig 1). Administratively, the watershed includes 73 villages across three towns-Chengliu, Sili, and Kejing-in Jiyuan City, covering a total area of 112.82 km^2^. The topography exhibits a distinct stepped pattern: the western part consists of medium-height mountains formed by erosion at the foothills of the Taihang Mountains, with elevations between 650 and 850 meters and a forest coverage rate of 78.2%; the central transitional zone is characterized by alluvial fans at elevations of 300–650 meters, where orchards and sloping farmland predominate; the eastern alluvial plain, at 150–300 meters above sea level, contains 82% of the region’s population and 91% of the industrial and mining enterprises. The climate is warm temperate continental monsoon, with an average annual precipitation of 567.9 mm, 72% of which which occurs during the rainy season from June to September.

### Soil sampling and laboratory analysis

To ensure regional soil environmental quality and agricultural product safety, the Henan Provincial Department of Natural Resources conducted a comprehensive soil sampling and analysis program throughout Jiyuan City. This initiative aims to provide a scientific basis for soil pollution remediation and ecological restoration. In accordance with the overall project design, this study focuses on a scientific analysis of sampling data from the Manghe River watershed. All soil samples were collected from publicly accessible areas, and no protected species were disturbed nor was any environmental damage caused during sampling activities. The data sources are legitimate and their use involves no ownership conflicts.

A 1 km × 1 km sampling grid was designed based on remote sensing and land use change survey data. Predefined sampling points were then optimized by taking into account factors such as traffic accessibility, water bodies, residential zones, industrial sites, topographic features, and field reconnaissance. A total of 121 soil sampling points were ultimately established (Fig 1). Sampling was conducted in March 2023, using a handheld GPS for precise positioning. At each point, five topsoil samples (0–20 cm) were collected according the plum-blossom sampling method. Approximately 1 kg of soil was then obtained from each composite sample by quartering and stored for laboratory analysis. Environmental conditions at each sampling site were documented in detail. In the laboratory, all samples were cleaned, air-dried, and finely ground to pass through a 100-mesh nylon sieve. They were then digested using a microwave-assisted four-acid procedure. Concentrations of Cr, Cu, Zn, and Ni were quantified by flame atomic absorption spectrometry(FAAS); Pb and Cd were analyzed using graphite furnace atomic absorption spectrometry(GFAAS); Hg was measured by cold vapor atomic absorption spectrometry (CVAAS), and As was qualified via atomic fluorescence spectrometry (AFS). All measurement were performed in triplicate, with standard deviation maintained within ±5% of the mean value. Quality assurance and control (QA/QC) was conducted using the certified soil reference material GBW07403 (GSS-3) obtained from the National Standardization Reference Materials Center of China. The relative standard deviation (RSD) was below 5%, and recoveries fell within ±10%, confirming the precision and reliability of the analytical results. Method detection limits were as follows: Cr 2 mg/kg, Hg 0.0003 mg/kg, As 0.05 mg/kg, Pb 1 mg/kg, Ni 1 mg/kg, Cd 0.02 mg/kg, Cu 1 mg/kg, and Zn 2 mg/kg. Outliers were identified and excluded using Grubbs’ test.

### Methodology

#### Semi-variance function model.

The semi-variance function model is a fundamental tool in geostatistics for characterizing spatial variability and has been widely utilized to investigate the spatial differentiation patterns of trace elements, soil physichemical properties, and heavy metal contamination [22].


$$\gamma (h)=\frac{\text{1}}{\text{2}N(h)}\sum_{i=\text{1}}^{N(h)}{\left[z(x_{i})-z(x_{i}+h)\right]}^{\text{2}}$$

whereas: γ(h) is the semi-variance function model; N(h) represents the total number of sample points when the segmentation distance is h; z(*x*_*i*_) denotes the measured value of the sample point at the spatial position x_i_; $\text{z}(x_{i}+\text{h})$ serves as the measured value of the sample point far away from h at *x*_*i*_.

#### Pollution Load Index.

The Contamination Factor (CF) method is a commonly used method for assessing the pollution level of individual heavy metal elements. To evaluate the overall extent of pollutant accumulation in environmental media such as soil, water, or atmosphere, the Pollution Load Index (PLI) was introduced by Roger Tomlinson et al. [23]. This index quantitatively assesses pollution severity by comparing the concentrations of multiple pollutants to their respective background values. Its calculation formula is as follow:


$${\text{CF}}_{\text{i}}=\frac{{\text{C}}_{\text{i}}}{{\text{C}}_{\text{n}}}$$

$$\text{PLI}=\sqrt[{\text{n}}]{{{\text{CF}}_{\text{1}}\times {\text{CF}}_{\text{2}}\times \cdots \times \text{CF}}_{\text{i}}}$$

whereas: *CF*_*i*_ is the pollution index of heavy metal *i*, *C*_*i*_ represents the test concentration of heavy metal *i*, *C*_*n*_ represents the evaluation criteria of heavy metals (here referring to the soil background value in Henan Province). *PLI* is the pollution load index of heavy metals, and *n* is the number of heavy metal elements. The classification of the pollution index is presented in Table 1.

**Table 1:** Classification of PLI.

CF	Pollution classification	PLI	Contamination level
≤0.7	Non-pollution	≤1	Non-pollution
0.7-1	Minor pollution	1-2	Light pollution
1-2	Light pollution	2-3	Moderate pollution
2-3	Moderate pollution	≥3	Heavy pollution
≥3	Heavy pollution		

#### PMF-PCA coupling model.

Principal Component Analysis (PCA) is a widely employed statistical technique for identifying potential sources of heavy metals in soil. Through dimensionality reduction, PCA elucidates the distribution patterns of heavy metals and provides the loadings (weights) of each metal on the principal component [24]. As a powerful receptor model, Positive Matrix Factorization (PMF) model analyzes the sample data matrix by decomposing it into two matrices: factor contributions and factor profiles. This method allows the incorporation of sample-specific uncertainties and enables weighted least-squares optimization for improved resolution of source apportionment [25]. The fundamental formula is expressed as:


$${\text{C}}_{\text{ij}}=\sum_{\text{k}=\text{1}}^{\text{P}}{\text{G}}_{\text{ik}}{\text{F}}_{\text{kj}}+\epsilon_{\text{ij}}$$

whereas: *C*_*ij*_ denotes the content of the *j*^*th*^ element in sample *i*, and *C*_*ik*_ indicates the contribution of the *k*^*th*^ pollutant source in sample *i*; *F*_*kj*_ signifies the eigenvalue of pollant source *k* to the *j*^*th*^ heavy metal concentration; *ℇ*_*ij*_ represents the residual, and *p* donates the number of factors. Based on the pollutant content and uncertainty data of the samples, a weighting coefficient is applied to derive the minimum objective function. The iterative minimization algorithm is then utilized to solve for *Q*, ensuring that under the condition of minimizing *Q*, the contribution rate of pollution sources and the pollution source component spectra are accurately calculated.


$$\text{Q}=\sum_{\text{i}=\text{1}}^{\text{n}}\sum_{\text{j}=\text{1}}^{\text{m}}\left(\frac{\epsilon_{\text{ij}}}{{\text{u}}_{\text{ij}}}\right)$$

whereas: *n* represents the number of samples, m denotes the number of pollutants, and *u*_*i*j_ is the uncertainty of heavy metals, which is determined as follows:


$${\text{u}}_{\text{ij}}=\frac{\text{5}}{\text{6}}\times \text{MDL}$$

$${\text{u}}_{\text{ij}}=\sqrt{(\text{EF}\times \text{c}{)}^{\text{2}}+(\text{0}.\text{5}\times \text{MDL}{)}^{\text{2}}}$$

whereas: *c* represents the heavy metal concentration, MDL denotes the elemental detection limit. The error fraction (EF) is typically assigned a value of 0.05 ~ 0.2; in this instance, it has been set at 0.1 [26].

## Results

### Descriptive statistics of soil heavy metals

The characteristics of heavy metal content in the topsoil of Manghe River watershed were summarized in Table 2. The findings indicated that the average pH was 7.73, indicating a neutral soil condition. The mean contents of Hg, As, Cr, Cu, Ni, Pb, Zn and Cd were 0.12 mg/kg, 19.2 mg/kg, 55.4 mg/kg, 31.9 mg/kg, 27.7 mg/kg, 95.2 mg/kg, 128 mg/kg, and 3.43 mg/kg, respectively. Among these, the concentration of Cr remained below the background level for soil in Henan Province. In contrast, the concentrations of Hg, As, Ni, Cu, Pb, Zn and Cd were 3.52, 1.69, 1.62, 1.04, 4.85, 2.14, and 46.4 times their respective background values, indicating significant enrichment-particularly of Hg, Pb, and Cd. The high kurtosis and skewness values for Cd and Hg further suggested pronounced accumulation of these two elements. The coefficient of variation (CV), a dimensionless measure of data dispersion relative to the mean, was used to assess variability in metal concentrations. Generally, CV ≤ 0.15 indicates weak variability, 0.15 < CV < 0.36 denotes medium variability, and CV ≥ 0.36 reflects strong variability [27,28]. The CV values for Cr and Ni were 0.17 and 0.19, respectively, indicating moderate variation. The remaining metals exhibited strong variability, with CV values descending as follows: Cd (3.05)> Hg (2.35)> Pb (1.49)> As (0.67)> Cu (0.64)> Zn (0.57). Notably, the exceptionally high CV values of Cd, Hg and Pb suggest significant anthropogenic influence and pronounced spatial heterogeneity.

**Table 2:** Descriptive statistics of soil heavy metal pollution.

Elemental	Sample size	Minimum	Maximum	Mean	Standard deviation	Variance	Kurtosis	Skewness	CV	Soil background
**Hg**	121	0.00	2.54	0.12	0.29	0.08	42.0	5.69	2.35	0.03
**As**	121	0.30	98.2	19.2	12.9	166	14.6	3.34	0.67	11.4
**Cr**	121	21.2	87.0	55.4	9.18	84.3	1.89	−0.17	0.17	63.8
**Cu**	121	0.00	179	31.9	20.4	417	23.4	4.04	0.64	19.7
**Ni**	121	9.87	42.3	27.7	5.21	27.1	1.11	−0.38	0.19	26.7
**Pb**	121	0.00	924	95.3	142	20000	16.7	3.76	1.49	19.6
**Zn**	121	32.2	534	128	72.8	5290	10.1	2.83	0.57	60.1
**Cd**	121	0.55	115	3.43	10.5	109	110	10.3	3.05	0.07
**pH**	121	4.94	8.53	7.73	0.63	0.39	5.12	−2.11	0.08	8.03

### Characteristics of heavy metal content in soils under different land use types

**Fig 2:**
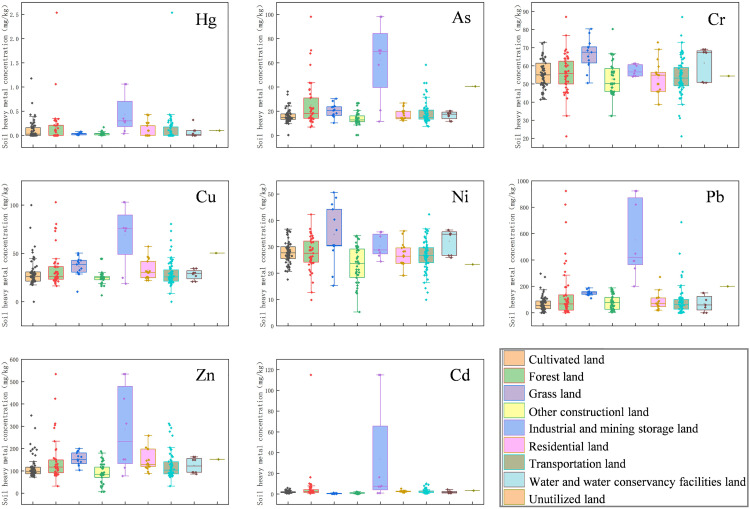
Box plots of heavy metal content on different land types.

As illustrated in Fig 2, the pollution characteristics of heavy metals varied markedly across different land use types. The data, which exhibited small minimal outliers, were highly reliable. While Cr and Ni exhibited a uniform distribution across various land use types, other heavy metals, particularly in industrial and mining areas, exhibited exceptionally high concentrations. Besides Cd, elements like Hg, As, Cu, Pb, and Zn also demonstrated notable distributions in cultivated land, forests, residential zones, and transportation corridors, underscoring the influence of human activities.

### Spatial distribution of soil heavy metals

#### Semi-variance model.

Table 3 presented the semi-variance model fitting results for eight heavy metals utilizing GS + 9.0. The coefficients of determination (R^2^) ranged from 0.672 and 0.802, all exceeding the threshold of 0.6, indicating a robust model fit. Specifically, Hg and Cu were best fitted by spherical models, As, Cr and Ni by exponential models, and Pb, Zn and Cd by Gaussian model. The nugget-to-sill ratio [C_0_/(C_0_ + C)] was used to evaluate the spatial dependence of each element. Ratios below 0.25 for Cr and Ni indicated strong spatial auto-correlation, implying that their distribution was primarily influenced by intrinsic factors such as soil structure and topography. For Hg, Pb, and Cd, the ratios fell between 0.25 and 0.75, suggesting moderate spatial auto-correlation influenced by both natural conditions and random factors. In contrast, As, Cu, and Zn exhibited ratios greater than 0.75, indicating weak spatial auto-correlation and a distribution dominated by stochastic processes and significant spatial variability.

**Table 3:** Parameters of the fitted semi-variational function model for soil heavy metals.

Variant	Theoretical model	Nugget (C0)	Sill (C0 + C)	C0/(C0 + C)	r2	Range
**Hg**	Spherical	0.091	0.115	0.774	0.791	2150
**As**	Exponential	0.185	0.522	0.354	0.802	2610
**Cr**	Exponential	0.084	0.342	0.246	0.696	1870
**Cu**	Spherical	0.044	0.082	0.537	0.756	1891
**Ni**	Exponential	0.375	1.710	0.219	0.778	2402
**Pb**	Gaussian	0.032	0.038	0.842	0.791	1506
**Zn**	Gaussian	1.342	2.090	0.642	0.672	1385
**Cd**	Gaussian	0.036	0.047	0.766	0.783	1470

#### Spatial distribution of heavy metals.

**Fig 3:**
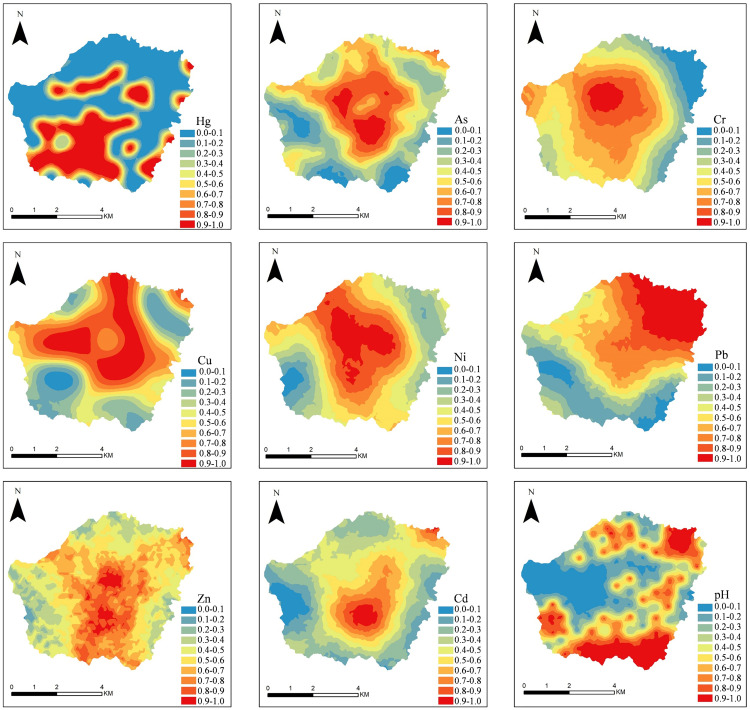
Distribution of soil heavy metal.

Fig 3 illustrated the spatial distribution of the eight heavy metals, as interpolated using indicator kriging. The spatial pattern of Cr and Ni were similar, both exhibiting a concentric structure with concentrations highest at the center and gradually decreasing toward the periphery in a regular ring-like pattern. The high-concentration zone extended from the northwest to the due south. Cu and As also exhibited similarity, with high values displaying a consistent circular gradient from the center to the periphery. Additionally, elevated values formed a distinctive “Y” shape in the northwest to southeast direction. The spatial distribution of Pb, Cd, and Zn shared general similarities, with high-value areas predominantly aligned along a northeast-to -southwest axis. Specific variations were observed: Pb reached its peak in the northeastern corner, with concentrations declining progressively toward the southwest; Cd showed high-value zones in the central and southern regions, whereas Zn showed a patchwork mosaic of high and low values concentrated in the mid-southern area. In contrast, Hg showed no discernible decreasing trend. Its high value region occurred as discrete, isolated patches within lower-concentration surroundings. Specifically, high-value zones in the southwest were contiguous, while those in the north were more fragmented and scattered.

### Evaluation of soil heavy metal pollution

#### Pollution load index method.

The pollution load indices of heavy metals was presented in Table 4. The *CF*_*i*_ decreased in the following order: Cd (30.35)> Pb (4.76)> Hg (3.62)> Zn (2.18)> As (1.76)> Cu (1.52)> Ni (0.99)> Cr (0.85). Based on these values, Cr and Ni exhibited minor pollution, As and Cu showed slight pollution, Zn demonstrated moderate pollution, while Hg, Pb, and Cd showed severe pollution. The overall mean Pollution Load Index (PLI) in the study area was 0.79, indicating a slight pollution level. The highest pollution indices for Hg, Cu, Pb, Zn and Cd were found in industrial and mining storage lands. Meanwhile, the pollution index of As peaked in unused land, followed by industrial and mining storage land. The pollution indices of Cr and Ni demonstrated consistent patterns across all land use types. The PLI values across different land use types descended in this order: industrial and mining storage land (6.60)> unused land (4.10)> forests (1.22)> other construction land (0.78)> residential land (0.70)> water bodies and water conservancy infrastructure (0.60) = grassland (0.60) = cultivated land (0.60)> transportation land (0.58). Notably, industrial and mining storage land and unused land were severely polluted, forests were slightly contaminated, and all other land use types remained within unpolluted levels.

**Table 4:** Heavy metal pollution load index under different site types.

Land use type	CF_Hg_	CF_As_	CF_Cr_	CF_Cu_	CF_Ni_	CF_Pb_	CF_Zn_	CF_Cd_	PLI
**Cultivated land**	1.64	1.46	0.83	1.47	0.93	3.41	1.96	17.52	0.60
**Forests**	4.36	2.29	0.85	1.64	0.98	7.39	2.47	53.17	1.22
**Grass land**	0.98	1.68	1.04	1.64	1.30	7.53	2.76	8.67	0.60
**Water and water conservancy facilities**	1.43	1.53	0.95	1.35	1.15	3.48	2.10	16.54	0.60
**Residential land**	3.01	1.59	0.82	1.57	0.96	4.23	2.58	22.69	0.70
**Industrial and mining storage land**	6.62	5.69	0.88	3.28	1.08	24.43	4.87	299.13	6.60
**Transportation land**	0.01	1.71	0.83	1.41	0.96	4.39	2.13	22.74	0.58
**Other constructionl land**	1.26	1.58	0.72	1.38	0.97	4.49	3.38	18.33	0.78
**Unused land**	1.42	6.46	0.84	2.62	1.03	19.66	2.57	64.42	4.10
**Study area as a whole**	3.62	1.76	0.85	1.52	0.99	4.76	2.18	30.35	0.79

### Sources apportionment of heavy metals

#### Correlation analysis.

Heavy metals derived from the same or similar sources typically exhibit strong correlations [29], as shown in Table 5. A highly significant positive correlation (p < 0.01) was observed between Cr and Ni, with a correlation coefficient of 0.887. Similarly, Pb, As, Cu, Zn, and Cd showed strong intercorrelations, with coefficient ranging from 0.517 to 0.827, suggesting that these elements likely originated from common or closely related sources. However, Hg showed consistently low correlation coefficient (below 0.354) with the other seven heavy metals, indicating a weak relationship and implying a distinct origin. Further investigation was required to elucidate the specific circumstances.

**Table 5:** Soil heavy metal correlation coefficients.

	Hg	As	Cr	Cu	Ni	Pb	Zn
**Hg**	1						
**As**	0.196*	1					
**Cr**	−0.115	0.148	1				
**Cu**	0.354**	0.497**	0.023	1			
**Ni**	−0.128	0.195*	0.887**	0.068	1		
**Pb**	0.216*	0.827**	0.023	0.517**	0.091	1	
**Zn**	0.266**	0.625**	−0.001	0.608**	0.030	0.703**	1
**Cd**	0.321**	0.686**	0.057	0.406**	0.137	0.673**	0.617**

** indicates 0.01 level of correlation, * indicates 0.05 level of correlation.

#### Principal component analysis.

The findings of the principal component analysis were presented in Table 6. The first principal component (PC1), explaining 44.01% of the total variance, exhibited high loadings for Pb, As, Zn, Cu, Cd, and Hg. Elevated concentrations of As, Pb, Cd, and Zn were observed in industrial and mining storage area, indicating a close link between these four heavy metals and industrial activities. Increased levels of Cd, Cu, and Zn were also detected in forests, grasslands, and cultivated lands, suggesting an association with agricultural practices. Hg was most abundant in industrial and mining storage land, with moderately high levels observed in forest and grassland soils. Its weak correlation with the other five heavy metals implied a distinct origin. PC1 was therefore primarily attributed to anthropogenic activities. The the second principal component (PC2) accounted for 25.38% of the variance, with strong loadings from Cr (0.954) and Ni (0.955). Given that previous analysis indicated Cr and Ni were mainly derived from parent material, PC2 was considered to represent natural sources.

**Table 6:** Principal component factor loadings.

Sports event	PC1	PC2
**Pb**	0.880	−0.032
**As**	0.868	0.111
**Zn**	0.828	−0.090
**Cu**	0.761	−0.062
**Cd**	0.757	−0.085
**Hg**	0.356	−0.291
**Ni**	0.141	0.955
**Cr**	0.092	0.954
**Variance contribution/%**	44.01	24.24
**Cumulative variance contribution/%**	44.01	68.25

#### Source apportionment by PMF.

**Fig 4:**
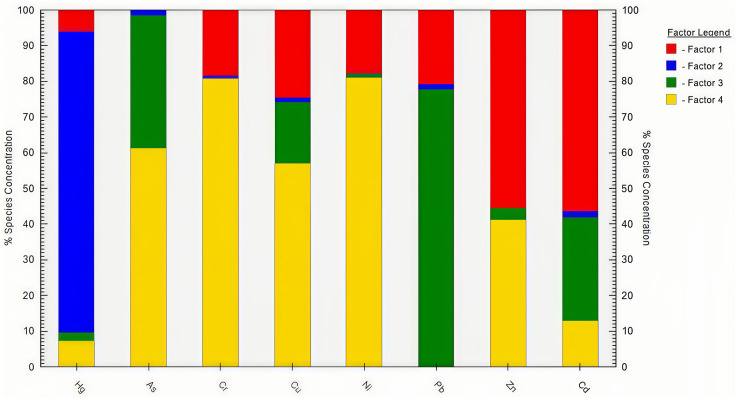
Source contributions of each factor generated by the PMF model.

Using EPA PMF 5.0 software, we analyzed the sources of eight heavy metals in the study area. A total of 3–6 factors were set, with 20 iterations performed to minimize analytical bias. Analysis results showed that when comparing different numbers of factors, setting 4 factors resulted in residual values predominantly distributed stably within the range of −3 to 3. Furthermore, the error between Qrobust and Qture did not exceed 25%. When running the PMF model with 4 factors, the R^2^ values for most elements exceeded 0.6, and the signal-to-noise ratios (S/N) for all elements were greater than 2.0. The category was set to “strong,” indicating that the PMF source apportionment model can meet the requirements for analyzing heavy metal sources in soil. The results of the source allocation are shown in Fig 4.

Factor 1 accounted for 34.95% of the total contribution. It was identified as the dominant source of Zn (62.1%) and Cd (60.3%). Given that Jiyuan City hosted Asia’s largest lead-zinc smelting base and possessed substantial zinc reserves, and considering that Cd was intrinsically linked to various industrial processes such as electroplating, metal smelting, and chemical production [30], Factor 1 was attributed primarily to industrial activities.

Factor 2 contributed 10.87%, with Hg accounting for 85% of its profile, while contributions to other metals each remained below 5%. Hg was commonly found in most minerals, and its concentration was amplified through mining and smelting operations. Additionally, coal combustion during smelting facilitated atmospheric deposition of Hg [31]. Thus, Factor 2 was interpreted as originating mainly from coal combustion.

Factor 3 explained 26.81% of the total variance and was a major contributor to Pb (80.95%) and As (35.76%). Traffic emissions, particularly from fuel combustion, engine wear, and catalyst utilization, constituted a primary source of Pb [32]. The exploitation of mineral resources and the improper disposal of industrial waste—such as smelting by-products and fossil fuel combustion residues—constituted major anthropogenic sources of As in soils. For instance, sulfide minerals like pyrite and oxide minerals such as hematite may contain As levels exceeding 100 g/kg [33]. Further more, As was commonly introduced via agricultural practices, such as the application of fertilizers (e.g., ammonium nitrate, ammonium phosphate, and compound fertilizers) and pesticides or herbicides containing inorganic arsenic [33]. Therefore, Factor 3 was considered a mixed source deriving from both transportation, industrial and agriculture activities.

Factor 4 contributed 27.37%, with high loadings of Cr (74.12%), Ni (74.61%), and Cu (50.84%). The contents of Cr and Ni were close to regional soil background values and exhibited low variability, suggesting a origin related to soil parent material and geological background [34–36]. In contrast, Cu was widely used in agricultural amendments such as pesticides, fertilizers and insecticides, like copper sulfate, copper oxide or copper carbonate, and livestock feed additives, leading to its accumulation in soils through manure application [37,38]. The elevated Cu content observed in cultivated land supported this inference. Hence, Factor 4 represented a mixed source influenced by both natural processes and agricultural practices.

## Discussion

### Spatial distribution heterogeneity of heavy metals

This study focuses on mixed soil samples collected from the top 0–20 cm layer, which serves as a critical interface for processes such as atmospheric dry and wet deposition and surface runoff erosion. This layer exhibits significant responses to environmental disturbances, including temperature, precipitation, and industrial thermal emissions [39], resulting in pronounced spatial heterogeneity in heavy metal concentrations. As shown in Figs 2 and 3, industrial and mining areas form distinct composite pollution hotspots of As, Pb, Zn, and Cd, with concentration levels significantly higher than those in other land use types. In contrast, cultivated land and forest-grassland show element-specific differentiation, supporting the value of topsoil as a “pollution process recorder” [40]. The concentration of Cr and Ni show a close relationship with land use types and topographic conditions in the study area. The terrain generally slopes from west to east, with forest areas dominating the west, cultivated land prevailing in the east, and various types of construction land concentrated in the central part. Although Cr and Ni pollution levels in Chinese soils are generally low [41], their distinct ring-like gradient pattern indicates significant anthropogenic interference. Specifically, open-air storage of chromite slag from Zn smelting operation, along with dispersion of Ni-based catalysts, has resulted in a diffusion pattern centered around the smelting plants via both dry and wet deposition, with concentrations decreasing radially outward. While Cu, As, Pb, and Cd also exhibit declining trends from high to low concentrations, their gradient directions and rates vary considerably, indicating different migration mechanisms. As displays a complex decreasing pattern, likely due to combined industrial and agricultural pollution. Cu decreases more uniformly, primarily associated with agricultural activities and population density. Pb, however, is strongly influenced by road networks, forming concentration peaks at major intersections. Hg exhibits a “nested island-type” distribution, influenced by multiple factors such as coal combustion emissions, land use and surface characteristics, monsoon climate effects, and historical pesticide residues [42].

### Source apportionment of heavy metals

This study employed correlation analysis, principal component analysis (PCA), and positive matrix factorization (PMF) to systematically identify the sources of heavy metals. Correlation analysis preliminarily revealed symbiotic relationships among elements, suggesting that highly correlated elements may share the same source or similar enrichment pathways [43,44]. PCA further categorized the pollution sources broadly into natural and anthropogenic origins. The PMF model then precisely identified and quantified the specific contributions of each pollution source. Capable of effectively handling missing and uncertain data, the PMF model yields source apportionment results that better reflect actual pollution characteristics [45]. Compared to traditional single-model approaches, the three-stage progressive analysis employed in this study demonstrates remarkable performance. It effectively addresses the resolution limitations of PCA for collinear pollution sources and accurately differentiates between mixed signals originating from coal combustion (Hg) and smelting processes (Cd-Zn). Furthermore, numerous studies have validated that integrating the PMF model with geostatistical methodologies provides a robust and practical strategy for pollution identification and supports the management of regional soil heavy metal contamination [46,47].

### Strategies for heavy metal pollution control

The development of chemical and smelting industries in Jiyuan City, coupled with intensive agricultural activities, constitutes the primary cause of soil heavy metal contamination in the region. In addition to Cr and Ni, all six other heavy metals exhibit varying degrees of accumulation, with Cd, Pb, and Hg showing notably elevated levels that warrant urgent attention. Especially, due to its high toxicity and mobility, mercury poses a potential threat to local residents through pathways such as inhaling dust and ingesting food from the food chain. Therefore, health screening must be intensified in pollution hotspots and strict source control measures must be implemented. The first step in controlling heavy metal pollution lies in enhancing monitoring efforts. Based on pollution load levels, a scientific zoning approach should be adopted to facilitate tailored soil utilization and remediation strategies according to contamination severity. Simultaneously, source analysis results should be integrated to enable targeted source control and end-of-pipe treatment. Jiyuan City has a high concentration of smelting and chemical industries, and coal combustion is a significant source of mercury pollution there. Therefore, comprehensive Hg removal throughout the coal combustion process is essential to reduce emissions. To mitigate As and Cu pollution from agricultural sources, modern fertilization techniques such as water-fertilizer irrigation, deep placement and band application should be intensified to enhance utilisation rates and reduce application quantities. For transportation-related Pb pollution, transitioning to clean energy alternatives instead of fuel oil is recommended. For industrial sources such as Zn and Cd, it is particularly crucial to strengthen production process controls to prevent element leakage and enhance waste management.

### Limitations and prospects

This study established a comprehensive technical framework based on ‘spatial differentiation, source analysis, and treatment response’. This offers a replicable and scalable “Jiyuan Model” for the prevention and control of heavy metal pollution in industrial and mining cities within the Yellow River Basin. However, given the multiple constraints such as human resources, material resources and funds, this study concentrated on the pollution characteristics and source analysis of heavy metals in the 0–20 cm soil surface over only a one-year period. The absence of data on atmospheric dry and wet deposition fluxes, water quality data (e.g., in rivers and groundwater) and long-term monitoring records resulted in insufficient information regarding the accumulation and migration of heavy metals under different land use types. Furthermore, the generalizability of our findings to other industrial and mining regions, particularly those with differing climatic regimes, industrial profiles, or hydrological settings, may be limited by these constraints. The lack of atmospheric dry and wet deposition flux data prevents a complete understanding of aerial input pathways, especially for highly volatile elements like Hg. The absence of complementary data on heavy metals in water bodies (e.g., rivers, groundwater) and sediments hinders the construction of a comprehensive cross-media migration model. Consequently, while the integrated methodological framework is proposed as a replicable ‘Jiyuan Model,’ its direct application to other areas requires caution and should be supported by region-specific data on atmospheric deposition, hydrology, and long-term environmental monitoring. Future research should focus on enhancing relevant studies in these areas. For instance, integrating a hydrological model like SWAT to simulate water and sediment runoff with a chemical transport model would be the most effective tool to quantitatively simulate the Hg migration pathways under monsoon conditions. Moreover, although the PMF model has been thoroughly validated as a robust and widely accepted tool for allocating heavy metal sources in soil, particularly when the number and distribution of potential sources is unknown, a single model cannot ultimately overcome its inherent limitations. For example, source attribution still relies on subjective judgement. Integrating PMF results with isotope fingerprinting technology can address this shortcoming and validate source identification.

## Conclusions

(1) The average concentrations of Cr and Ni in the study area were close to the background values of soils in Henan Province, while Hg, As, Cu, Pb, Zn, and Cd all exhibited varying degrees of enrichment, particularly pronounced in industrial and mining storage land. Hg, Pb and Cd contamination was especially severe, exceeding their background values by factors of 3.52, 4.85 and 46.4, respectively.
(2) The spatial distribution patterns of the heavy metals were distinct: Cr and Ni decreased concentrically from central high-value zones toward the periphery in a regular annular gradient. Although Cu, As, Pb and Cd also demonstrated decreasing trends from high to low values, the gradient directions and rates differed. Hg exhibited a discrete ‘high-low nested’ distribution pattern. Overall, the spatial differentiation of heavy metals correlated strongly with land use types and clearly reflects anthropogenic influences.
(3) Evaluation using the Pollution Load Index (PLI) classified Cr and Ni as slightly polluted, As and Cu as mildly polluted, Zn as moderately polluted, and Hg, Pb, and Cd as heavily polluted. The regional average PLI indicated mild pollution overall.
(4) Source apportionment analysis revealed significant correlations between Cr and Ni, as well as among As, Cu, Pb, Zn and Cd. In contrast, Hg exhibited weak correlations with other elements. PCA showed that Hg, As, Cu, Pb, Zn and Cd originated primarily from human activities, while Cr and Ni were mainly derived from natural sources. The PMF model further identified specific sources: Cr and Ni were influenced by soil parent material; Hg was attributed to coal combustion; Zn and Cd predominantly came from industrial emissions; Cu and As arose from mixed industrial-agricultural sources; and Pb was mainly associated with transportation emissions.

## Supporting information

S1 File Supplementary material. (ZIP)
